# Clonazepam-induced lichenoid drug eruption: a case report

**DOI:** 10.1186/s12888-021-03132-2

**Published:** 2021-03-04

**Authors:** Hee Won Yang, Jong Bin Bae, Jung-Im Na, Ki Woong Kim

**Affiliations:** 1 Department of Neuropsychiatry, Seoul National University Bundang Hospital, Seongnam, South Korea; 2 Department of Psychiatry, Seoul National University, College of Medicine, Seoul, South Korea; 3 Department of Dermatology, Seoul National University Bundang Hospital, Seongnam, South Korea; 4 Department of Brain and Cognitive Sciences, Seoul National University College of Natural Sciences, Seoul, South Korea

**Keywords:** Clonazepam, Cutaneous, Lichenoid, Drug eruption

## Abstract

**Background:**

Lichenoid drug eruption is rare and can mimic idiopathic lichen planus and other dermatoses. Clonazepam, a commonly used drug for the treatment of anxiety-related disorders and seizures, is known to be an unlikely cause of cutaneous adverse effects. Only one case report of LDE due to clonazepam has been reported.

**Case presentation:**

A 81-year-old male patient with Alzheimer’s disease developed a lichenoid eruption after taking clonazepam. He developed a violaceous scaly patch on his lower extremities, from both buttocks to the feet. The cutaneous eruption resolved 2 months after cessation of clonazepam and with initiation of corticosteroid therapy.

**Conclusion:**

A skin eruption that develops after clonazepam administration can be a lichenoid drug eruption, which is less likely to resolve spontaneously and requires discontinuation of clonazepam administration.

## Background

Drug eruption is the most common adverse drug reaction. The list of conditions that can be triggered by medications includes nearly all dermatological diseases, ranging from mild and moderate such as pruritus and lichenoid eruptions to severe ones like Stevens-Johnson syndrome [1]. Lichenoid drug eruption (LDE) is a rare type of drug eruption that can resemble idiopathic lichen planus (LP) clinically and histopathologically. LDE is induced by various drugs, such as gold salts, antimalarial drugs, β-blockers, angiotensin-converting-enzyme inhibitors [2]. Clonazepam is one of the most commonly used drugs for treating anxiety-related disorders and seizures. Common side effects such as drowsiness and sedation are well known and can be appropriately considered by clinicians. However, rare hypersensitivity reactions and cutaneous adverse effects are difficult to consider in most clinical situations and only one case report of LDE due to clonazepam has been reported [3, 4].

In the current case report, we present an 81-year-old Alzheimer’s disease (AD) patient with LDE induced by clonazepam. We aim to increase the clinicians’ insights into a rare drug eruption, and importance of early identification of the offending drug which may reduce unnecessary discomfort in patients due to delayed diagnosis.

## Case presentation

An 81-year-old man with 3 to 4 years of slowly progressive memory impairment visited the clinic for evaluation. The patient reported difficulty in recalling where he placed objects, and difficulty in understanding news programs. Except for memory impairment, other cognitive domains such as language, orientation, judgment, personality, and behavior were intact. In neuropsychological tests, he showed poor performance in word list recall and recognition, below − 2 standard deviation, while the scores in other cognitive domains were within the normal limit. He was receiving long-term treatment with enalapril for hypertension and had no other diseases or medications. He had a history of regular alcohol consumption (2 standard drinks per day) for 56 years, and had quit smoking 30 years ago. Detailed physical and neurological examinations did not reveal any abnormality. His blood tests were normal except for mild hypercholesterolemia. Magnetic resonance imaging (MRI) showed diffuse brain atrophy and bilateral hippocampal atrophy. Medial temporal lobe scores were 1/2 each on the left and right according to the Skeltens scale [5]. According to the Fazekas scale, the rating of white matter hyperintensities was 2 on the periventricular white matter and 1 on the deep white matter [6]. A positron emission tomography (PET) scan showed diffuse hypometabolism in the left frontoparietal cortices. Based on clinical findings and imaging biomarkers, he was diagnosed with mild cognitive impairment due to AD according to the diagnostic criteria proposed by the National Institute on Aging-Alzheimer’s Association (NIA-AA) [7]. Donepezil was started at 2.5 mg/day, escalating to 10 mg/day over several months. At 18 months, he had difficulty with nighttime sleep, and clonazepam 0.25 mg/day was started.

**Fig. 1:**
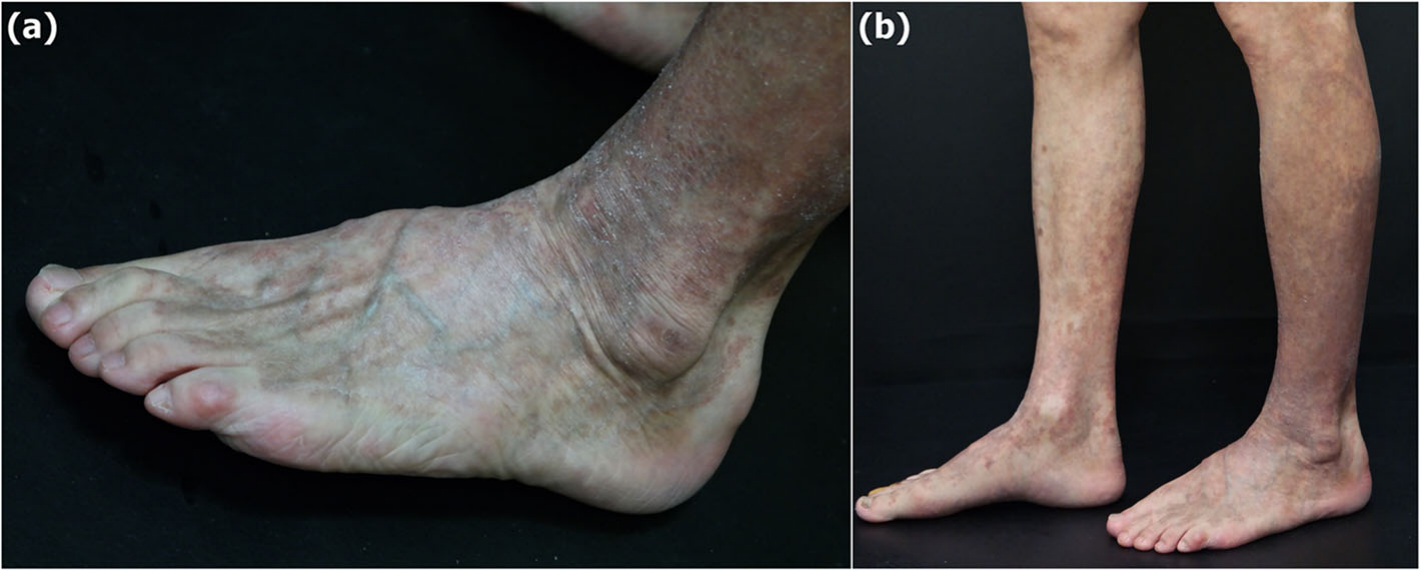
Violaceous scaly patch on the left foot (**a**) and both lower legs (**b**)

**Fig. 2:**
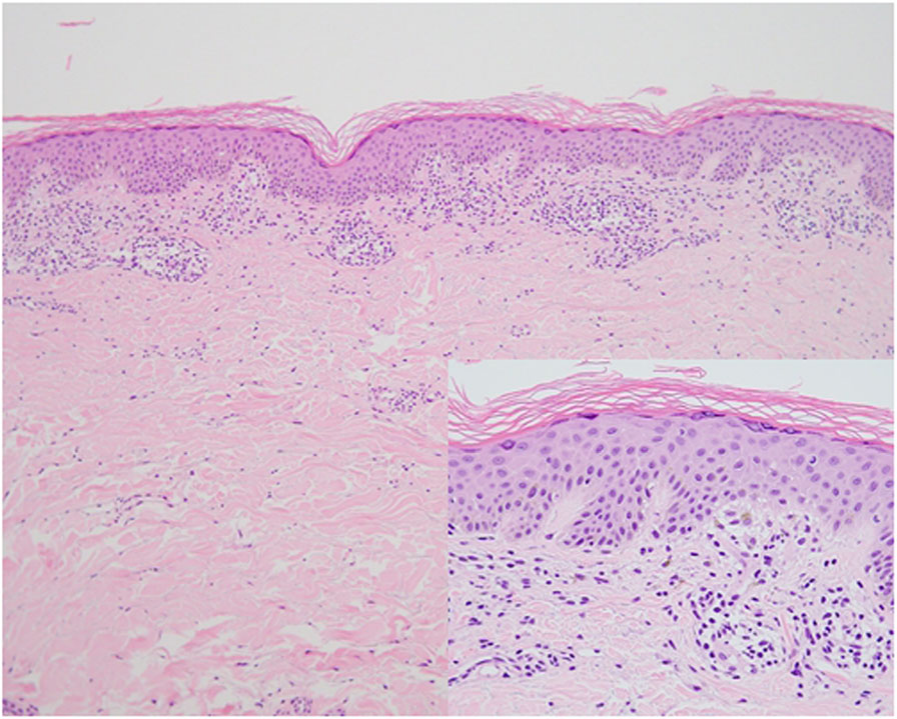
Histopathological findings show lichenoid dermatitis pattern with superficial perivascular lymphocyte infiltration and focal basal vacuolization (H&E stain, × 100, × 400)

Three weeks after starting clonazepam, the patient visited the dermatology outpatient clinic in our hospital with itchy eruptions on the lower extremities. His skin lesions had already improved to some extent by self-applying steroid ointment 3 days before. Because he had suffered from unspecified dermatitis for a long time and there was no history of allergy or drug reaction, a drug eruption was not suspected at the time. Since the lesions started to resolve, he was decided to follow up without special treatment, and only a p.r.n. topical steroid was prescribed. Since then, the lesions did not fully remit but rather spread, and the patient re-visited after 4 months. Physical examination revealed a violaceous scaly patch on the lower extremities, from both buttocks to feet (Fig. 1a,b). For further evaluation, laboratory tests and tissue biopsy were performed. His eosinophil count was slightly elevated to 7.1%, while other laboratory values were within the normal range. The histopathological findings of the eruptions on the left lower leg showed a lichenoid dermatitis pattern (Fig. 2). Based on clinical and histopathological findings, clonazepam-induced LDE was most strongly suspected. Clonazepam was discontinued, and zolpidem was prescribed for sleep control. Additionally, he was treated with a week of oral steroids and tacrolimus 0.1% ointment.

Although new lesions appeared on the upper extremities, most of the existing lesions gradually improved 1 month after clonazepam was discontinued. All eruptions disappeared over the next month and there was no recurrence after that. Sleep was well controlled by intermittent administration of zolpidem, and no more benzodiazepine was prescribed, including clonazepam. Memantine was added due to progressive cognitive decline, but no adverse reaction occurred.

## Discussion and conclusions

In the current case, LDE developed in patients with preexisting dermatitis after taking clonazepam, and it was improved over several months after cessation of the drug. Clonazepam is commonly prescribed for treating a range of disorders for which it had not originally been approved, including sleep-related disorders, withdrawal from other benzodiazepines, and pain management [8]. Clonazepam has been rarely associated with cutaneous adverse reactions, and only a few cases, such as erythema multiforme, pseudo-mycosis fungoides, localized exfoliating eruptions, alopecia, bullous dermatosis, and eczematous drug eruption have been reported [9–14]. To the best of our knowledge, only two case reports of LDE due to clonazepam has been reported [4, 15].

Cutaneous adverse effects of clonazepam are idiosyncratic and have been reported in a few patients taking various doses ranging from 0.5 to 2 mg/day [9–14]. Only one patient developed an LDE with clonazepam; however, this patient’s dosage was not stated [4]. Our patient with clonazepam-induced lichenoid eruption was treated at 0.25 mg/day. The incidence of LDE was known to be dependent on the causative drug rather than the dose [2]. LDE is less likely to resolve spontaneously and may require cessation of the offending agent in addition to corticosteroid therapy [16]. Therefore, in the case of clonazepam-induced LDE, it may be reasonable to discontinue the drug rather than to consider dose reduction.

LDE seems to have a long latent period of 4 weeks to 3 years, while the latent period of most drug eruptions is about 1 or 2 weeks, or up to 1 month. The identification of the offending drug can be complicated by such variability in the latent period between intake of a drug and appearance of the eruption. The latent period may vary depending on the offending drug, the dosage of the drug, and the patient’s individual reaction to the drug. The length of the resolution also depends on the offending drug, and it may be up to 5 months [2]. In our case, it was difficult to determine the onset of LDE due to comorbid dermatitis, but the latent period may be estimated from about 3 weeks to 4 months. After clonazepam was discontinued, it took 2 months for the lesions to disappear, which was in line with the typical course of LDE.

In the current case, the patient has also taken enalapril and lichen planus-like eruptions induced by enalapril have been reported a few times in the literature [17–19]. The contribution of clonazepam and enalapril in our patient**’**s development of LDE was evaluated by using the Naranjo Adverse Drug Reaction (ADR) Probability Scale [20], which revealed the probable influence of clonazepam and the possible influence of enalapril in LDE development. Therefore, it may be presumed that clonazepm had a higher impact on LDE development than enalapril. In addition, previous literatures reported that lichen planus-like eruption due to enalapril appeared between 6 weeks and 6 months [17–19]. In our case, enalapril had been taken for 11 years, which was less likely to cause LDE because it is much longer than the longest reported latent period.

Cytochrome P-450 including CYP3A, may play an important role in clonazepam metabolism. There is no evidence that clonazepam induces its own metabolism or that of other drugs in humans [3]. Enalapril is prodrugs metabolized in the liver, and some animal studies provide information suggesting that prodrugs seem to undergo CYP3A4-dependent biotransformation. However, the angiotensin-converting enzyme inhibitors including enalapril are not involved in significant cytochrome P450–mediated interactions with other drugs [21]. Therefore, a clinically significant interaction was not strongly expected.

Similar to other forms of drug eruptions, withdrawal of the suspected drug followed by the disappearance of the lichenoid eruption may confirm the diagnosis of LDE [2]. In this case, the course of rash disappearance after cessation of clonazepam was clear, so it was possible to diagnose clonazepam-induced LDE. As in the previously reported case, the most confirmative way to determine the causative drug may be to ascertain the disappearance of the eruption after cessation of the suspected drug and to reproduce the eruption by re-administering the drug [4]. However, re-exposure of the offending drug for diagnosis only may be dangerous and, therefore, not recommended. Topical provocation tests, which were not performed in our case, are safer, while it is known to have a higher false-negative rate [2, 22].

In conclusion, although it is not frequent, clonazepam may cause drug eruptions, such as LDE. Therefore, if eruptions appear after newly prescribed clonazepam, a thorough history taking and investigations should be performed, and if necessary, changing the drug and referencing to a dermatologist are advised.

## Data Availability

Not applicable.

## Abbreviations

LDE: Lichenoid drug eruption
LP: Lichen planus
AD: Alzheimer’s disease
MRI: Magnetic resonance imaging
PET: Positron emission tomography
NIA-AA: National Institute on Aging-Alzheimer’s Association
